# Genome Wide Meta-analysis Highlights the Role of Genetic Variation in *RARRES2* in the Regulation of Circulating Serum Chemerin

**DOI:** 10.1371/journal.pgen.1004854

**Published:** 2014-12-18

**Authors:** Anke Tönjes, Markus Scholz, Jana Breitfeld, Carola Marzi, Harald Grallert, Arnd Gross, Claes Ladenvall, Dorit Schleinitz, Kerstin Krause, Holger Kirsten, Esa Laurila, Jennifer Kriebel, Barbara Thorand, Wolfgang Rathmann, Leif Groop, Inga Prokopenko, Bo Isomaa, Frank Beutner, Jürgen Kratzsch, Joachim Thiery, Mathias Fasshauer, Nora Klöting, Christian Gieger, Matthias Blüher, Michael Stumvoll, Peter Kovacs

**Affiliations:** 1 Department of Medicine, University of Leipzig, Leipzig, Germany; 2 Institute of Medical Informatics, Statistics and Epidemiology, University of Leipzig, Leipzig, Germany; 3 LIFE Research Center, University of Leipzig, Leipzig, Germany; 4 IFB Adiposity Diseases, University of Leipzig, Leipzig, Germany; 5 Research Unit of Molecular Epidemiology, Helmholtz Center Munich, German Research Center for Environmental Health, Neuherberg, Germany; 6 Institute of Epidemiology II, Helmholtz Center Munich, German Research Center for Environmental Health, Neuherberg, Germany; 7 German Center for Diabetes Research (DZD e.V.), Neuherberg, Germany; 8 Department of Clinical Sciences, Diabetes and Endocrinology, Lund University and Lund University Diabetes Centre, CRC at Skåne University Hospital, Malmö, Sweden; 9 Department for Cell Therapy, Fraunhofer Institute for Cell Therapy and Immunology, Leipzig, Germany; 10 Institute of Epidemiology II, Helmholtz Zentrum München, German Research Center for Environmental Health, München, Germany; 11 Institute of Biometrics and Epidemiology, German Diabetes Center, Leibniz Center for Diabetes Research at Heinrich Heine University Düsseldorf, Düsseldorf, Germany; 12 Wellcome Trust Centre for Human Genetics, University of Oxford, Oxford, United Kingdom; 13 Oxford Centre for Diabetes, Endocrinology and Metabolism, University of Oxford, Oxford, United Kingdom; 14 Department of Genomics of Common Diseases, Imperial College London, London, United Kingdom; 15 Department of Social Services and Health Care, Jakobstad, Finland; 16 Folkhälsan Research Centre, Helsinki, Finland; 17 Institute of Laboratory Medicine, Clinical Chemistry and Molecular Diagnostics, University Hospital Leipzig, Leipzig, Germany; 18 Institute of Genetic Epidemiology, Helmholtz Zentrum München, German Research Center for Environmental Health, Neuherberg, Germany; Wellcome Trust Sanger Institute, United Kingdom

## Abstract

Chemerin is an adipokine proposed to link obesity and chronic inflammation of adipose tissue. Genetic factors determining chemerin release from adipose tissue are yet unknown. We conducted a meta-analysis of genome-wide association studies (GWAS) for serum chemerin in three independent cohorts from Europe: Sorbs and KORA from Germany and PPP-Botnia from Finland (total N = 2,791). In addition, we measured mRNA expression of genes within the associated loci in peripheral mononuclear cells by micro-arrays, and within adipose tissue by quantitative RT-PCR and performed mRNA expression quantitative trait and expression-chemerin association studies to functionally substantiate our loci. Heritability estimate of circulating chemerin levels was 16.2% in the Sorbs cohort. Thirty single nucleotide polymorphisms (SNPs) at chromosome 7 within the retinoic acid receptor responder 2 (*RARRES2)/Leucine Rich Repeat Containing (LRRC61)* locus reached genome-wide significance (p<5.0×10^−8^) in the meta-analysis (the strongest evidence for association at rs7806429 with p = 7.8×10^−14^, beta = −0.067, explained variance 2.0%). All other SNPs within the cluster were in linkage disequilibrium with rs7806429 (minimum r^2^ = 0.43 in the Sorbs cohort). The results of the subgroup analyses of males and females were consistent with the results found in the total cohort. No significant SNP-sex interaction was observed. rs7806429 was associated with mRNA expression of *RARRES2* in visceral adipose tissue in women (p<0.05 after adjusting for age and body mass index). In conclusion, the present meta-GWAS combined with mRNA expression studies highlights the role of genetic variation in the *RARRES2* locus in the regulation of circulating chemerin concentrations.

## Author Summary

Chemerin is an adipokine proposed to link obesity and chronic inflammation of adipose tissue. In the present study we show that circulating chemerin is a heritable trait. In a meta-analysis of genome-wide association studies (GWAS) of 2,791 individuals from Germany and Finland, we identified common genetic variants which associate with serum chemerin levels. The variants map within the retinoic acid receptor responder 2 (*RARRES2)/Leucine Rich Repeat Containing (LRRC61)* at chromosome 7. To better understand the potential functionality of the identified variants, we also provide insights into the mRNA expression of *RARRES2* (encoding chemerin) in blood and adipose tissue. Our results highlight the role and function of genetic variation in the *RARRES2* locus in the regulation of circulating chemerin concentrations.

## Introduction

Chemerin has been extensively studied as an adipokine associated with obesity and related phenotypes [1]–[4]. It is secreted from adipose tissue as an 18-kDa precursor protein which is activated by several extracellular cleavage steps [5]–[7]. The class of proteases responsible for the transformation of pro-chemerin to chemerin also determines pro-inflammatory or anti-inflammatory function of the protein. Interestingly, proteolytic processing is also suggested to be involved in the inactivation of the protein. By binding with the G protein-coupled receptor chemokine-like receptor 1 (CMKLR1) chemerin activates nuclear factor-kB and MAPK pathways [1], [6], [7].

Chemerin is highly expressed in white adipose tissue and its expression and secretion increases with adipogenesis [1], [5]. From a physiological point of view, chemerin was initially reported as a chemo-attractant for several types of immune cells [6]. Because of its role in chemotaxis of dendritic cells and macrophages, this adipokine is proposed to be a critical link between obesity and chronic inflammation of adipose tissue. Serum chemerin concentrations have been shown to be moderately heritable, with about 25% of variation attributed to genetic factors [8]. A recent genome-wide association analysis (GWAS) in 523 Mexican-American individuals revealed 7 loci moderately associated with chemerin serum concentrations. However, none of the suggested variants achieved genome-wide significance level and a replication cohort was not available in that study [8]. The single nucleotide polymorphism (SNP) showing the strongest evidence of association (rs347344; p = 1.4×10^−6^) was located within epithelial growth factor-like repeats and discoidin I-like domains 3 (*EDIL3*) playing an important role in angiogenesis, a key step in adipose tissue expansion in obesity [8].

Notably, variants in retinoic acid receptor responder 2 (*RARRES2*), the gene encoding chemerin, have been shown to be associated with increased visceral fat mass in non-obese subjects [9] and with increased incidence of the metabolic syndrome [10]. Co-expression network analyses in gluteal and abdominal adipose tissue showed that rs10282458 in the *RARRES2/REPIN1* region modulated *RARRES2* expression and was associated with body mass index [11].

In summary, none of the previously reported loci has either reached genome-wide significance levels or has been sufficiently replicated. Thus, the heritability of serum chemerin concentration still remains largely unexplained. Therefore, we conducted a meta-analysis of GWAS for serum chemerin in three independent cohorts: the Sorbs (N = 824) and KORA (N = 1630) from Germany and the PPP-Botnia (N = 337) from Finland.

To functionally support our GWAS findings, we performed more detailed analyses in the Sorbs cohort comprising interaction, gene expression quantitative trait loci, expression–association and causal analyses of peripheral blood mononuclear cells (PBMC) mRNA expression profiles of genes mapping within the associated loci as well as Gene expression analyses in adipose tissue by quantitative RT-PCR in an independent sample.

## Results

### Association of serum chemerin concentrations with obesity and parameters of glucose metabolism in the Sorbs cohort

Serum chemerin concentrations were positively correlated with BMI (p = 2.4×10^−10^, beta 0.442) and % body fat (p = 7.9×10^−9^, beta = 0.008) but not with fat distribution (Table 1). Furthermore, chemerin levels correlated with elevated fasting insulin (p = 3.5×10^−4^, beta = 0.071), HOMA-IR (p = 0.001, beta = 0.065) and AUC_glucose_ (p = 0.033, beta = 0.145). Interestingly, serum chemerin concentrations are also positively associated with adipocyte fatty acid binding protein (AFABP) and progranulin levels but not with vaspin and adiponectin concentrations (Table 1). Additionally, chemerin levels were negatively correlated with renal function (p = 7.8×10^−5^, beta = −0.255).

**Table 1:** Correlation of serum chemerin concentrations with anthropometric parameters and markers of glucose metabolism, serum lipids and renal function in the Sorbs cohort.

Trait	*p*-value	*ß*
**%body fat**	**7.9×10^−9^**	0.008
**BMI**	**2.4×10^−10^**	0.442
**WHR (adj. age, sex)**	**0.004**	0.415
**WHR (adj. age, sex, BMI)**	0.861	0.028
**fasting insulin**	**3.5×10^−4^**	0.071
**HOMA_IR**	**0.001**	0.065
**Stumvoll Index 1**	**0.015**	−0.119
**Stumvoll Index 2**	**0.002**	−0.177
**AUC glucose**	**0.033**	0.145
**AFABP**	**4.1×10^−11^**	0.006
**progranulin**	**9.7×10^−6^**	0.181
**vaspin**	0.344	0.01
**adiponectin**	0.49	0.023
**LDL cholesterol**	**0.021**	0.089
**GFR (MDRD)**	**7.8×10^−5^**	−0.255

Associations were assessed in a linear regression model adjusting for age, sex and BMI (except for analysis of BMI itself). HOMA-IR was calculated as published by Matthews et al. [35]. The Stumvoll Index refers to Stumvoll ISI_(0 and 120 min) and_ Stumvoll ISI_(0 and 30 min)_ [36]. Glomerular filtration rate (GFR) was calculated using the MDRD formula. *ß* Beta coefficient of linear regression model.

### Re-estimation of the heritability of chemerin concentration

The significant degree of relatedness observed in the Sorbs cohort allowed us to estimate the heritability of chemerin concentration, which was 16.2%, using a mixed-model approach proposed by Amin et al. and Aulchenko et al. [12], [13].

### Genome wide meta-analysis for association with serum chemerin concentrations

**Figure 1:**
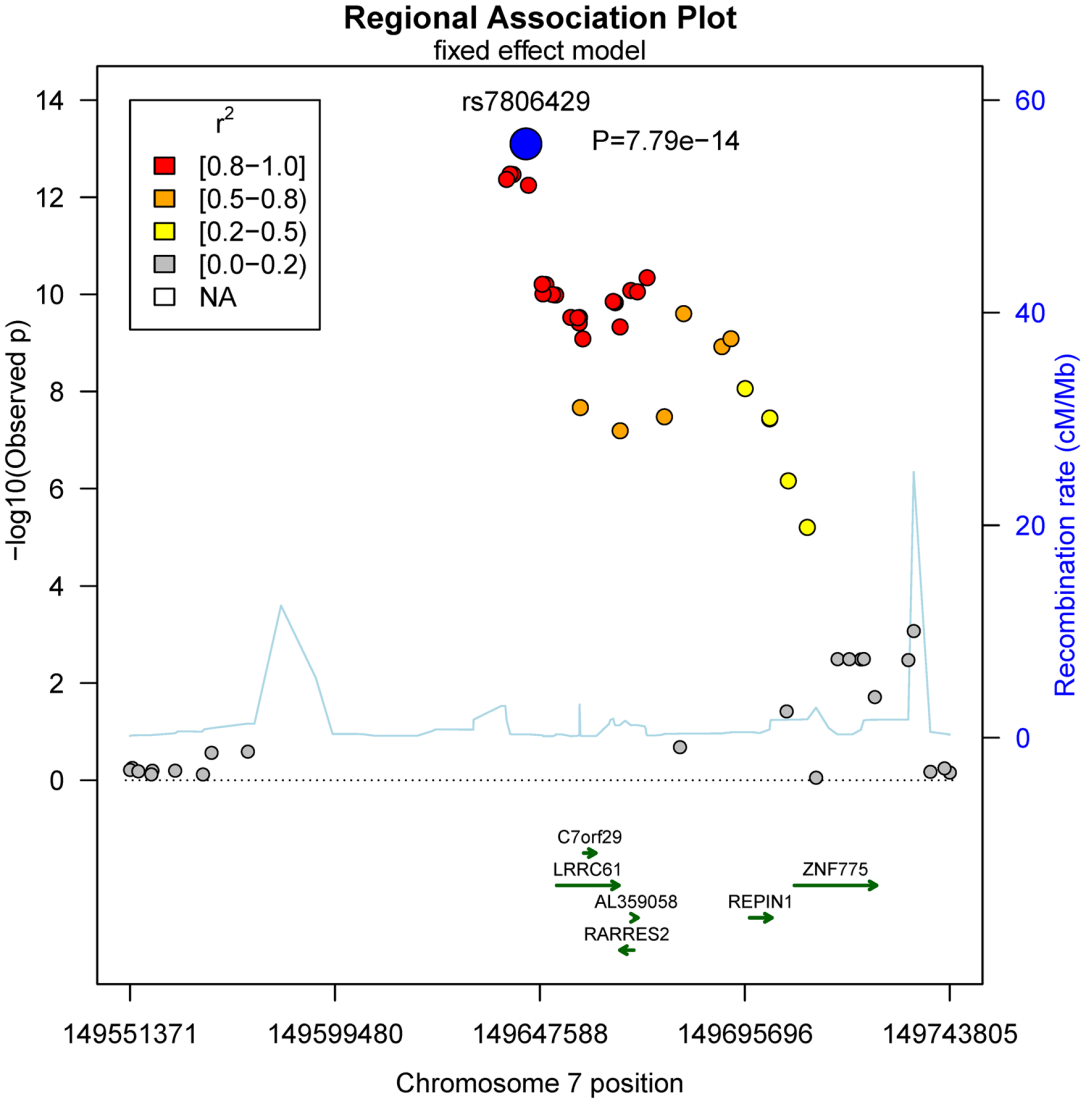
Regional association plot for the top-hit of association with chemerin levels on chromosome 7.

No general inflation of meta-analysis statistics was observed (λ = 0.99). Based on the fixed effects model, 30 SNPs on chromosome 7 reached genome-wide significance (p<5.0×10^−8^) in the meta-analysis of Sorbs (n = 824), KORA (n = 1630) and PPP (n = 337, Table 2). All of these SNPs were consistently associated with p<10^−3^ in both the KORA and the Sorbs cohorts but not in the PPP cohort. However, effect sizes and directions of effects were concordant between all three studies. The strongest evidence for association was observed for rs7806429 at chromosome 7 (p = 7.8×10^−14^, beta = −0.067, explained variance 2.0%, Table 2 and S1 Figure). All other SNPs with p<5.0×10^−8^ were in linkage disequilibrium (LD) with rs7806429 (minimal r^2^ = 0.43 in the Sorbs cohort). When adjusting chemerin levels for rs7806429, these SNPs are no longer significant in the Sorbs cohort (minimal p-value 0.19), i.e. no independent effects of other SNPs at this locus were detected. A regional association plot is provided in Fig. 1. The obvious gap detectable between positions 149.58 Mbp and 149.64 Mbp is caused by low imputation efficacy of Affymetrix 500 K SNP panels resulting in post-imputation quality drop-outs in the Sorbs cohort and in the Botnia study.

**Table 2:** SNPs associated with circulating chemerin levels at genome-wide significant levels.

				Alleles		Combined – fixed effect model			Sorbs		KORA		PPP-Botnia	
Nearby Gene	Lead SNP	Position (Chr. 7)	SNPs in LD (r2>0.8)	major	minor	effect	SE	*p*-value	*p*-value	*β*	*p*-value	*β*	*p*-value	*β*
*ACTR3C*	rs7806429	149644326	rs17837498,	T	C	−0.067	0.009	7.79×10^−14^	2.48×10^−5^	−0.077	6.87×10^−10^	−0.071	0.17	−0.032
			rs11975886,											
			rs7781827,											
			rs7788316,											
			rs6964110,											
			rs2159236,											
			rs11767726,											
			rs17173617,											
			rs3735167,											
			rs2108854,											
			rs2108852,											
			rs1962004,											
			rs3735171,											
			rs3735170,											
			rs3735169,											
			rs1983440,											
			rs10952250,											
			rs4554381,											
			rs11764936,											
			rs1047575,											
			rs11769348											
*RARRES2/REPIN1*	rs10259796	149681360	rs7800196,	T	C	−0.059	0.009	2.48×10^−10^	2.80×10^−4^	−0.071	3.02×10^−8^	−0.066	0.593	−0.012
			rs17173681											
*REPIN1*	rs6946097	149695799	rs1051764,	T	C	0.050	0.009	8.67×10^−9^	3.44×10^−5^	0.073	4.04×10^−6^	0.052	0.796	0.006
			rs1051760											
*RARRES2*	rs9640161	149676843	rs10247016	C	A	−0.048	0.009	3.30×10^−8^	3.54×10^−4^	−0.066	1.26×10^−5^	−0.047	0.457	−0.018

Inflation factor of all single studies were negligible (maximum λ = 1.014 in the Sorbs cohort). No inflation of meta-analysis results was observed (λ = 0.99). Positions of SNPs are given according to HapMap2 CEU, Release 24, dbSNP-build 126, NCBI 36 as the reference panel. Effect directions are given for the coding allele. *ß* Beta coefficient of regression model.

Chr: chromosome; *β*: beta; SE: standard error; *LRRC61*: leucine rich repeat containing 61; *ACTR3C*: ARP3 actin-related protein 3 homolog C; *RARRES2*: retionic acid receptor responder 2; *REPIN1*: replication initiator 1; *ZBED6CL*: ZBED6 C-terminal like.

The results of the subgroup analyses in males and females were consistent with data in the total cohort (S2 Table). No significant interaction of rs7806429 and sex was observed (p = 0.83 in the Sorbs cohort).

Of note, 4 SNPs not in LD with rs7806429 have been identified with p-values<10^−6^ using the fixed effect model in at least one of the groups “all”, “male” or “female” (S2 Table). We consider these results as being suggestive but requiring further investigations. Thus, two other loci on chromosomes 3 (lead SNP: rs2594989 in *ATG7* - Autophagy Related 7) and 15 (lead SNP: rs8027521) should be considered. The first locus showed consistent effects across all studies and subgroups but failed to reach genome-wide significance. The significant effect of the locus on chromosome 15 was primarily due to the strong effect size observed in the Sorbs cohort (S2 Table).

We analysed the correlation of our top-hits with published GWAS-hits of the GWAS catalogue (downloaded on 12th Sept., 2013). The maximum r^2^ was 0.2 for the hit on chromosome 15, suggesting there are no pleiotropic effects regarding published GWAS traits [14].

Finally, we performed a Mendelian randomization analysis using our top-SNP as an instrumental variable. We considered different parameters of obesity or the metabolic syndrome as possible causal endpoints of chemerin (Table 1). Results are presented in S6 Table. Analysis suggests a causal relationship of chemerin and adiponectin while causality regarding all other endpoints was not significant.

### Replication of association signals in the LIFE Leipzig Heart Study

Replication of top-SNPs was performed in 967 samples of the LIFE Leipzig Heart Study [15]. All top-SNPs were either directly measured or imputed with high quality, i.e. no proxies were required. Replication analysis revealed a clear support of our results at chr. 7 with similar effect sizes as observed for the Sorbs and KORA cohort (S7 Table). In contrast, no additional evidence was generated for the loci at chrs. 3 and 15.

### mRNA expression studies

#### PBMCs

Using mRNA expression data obtained by genome-wide micro-arrays, we performed an eQTL analysis in PBMCs for rs7806429 in the Sorbs cohort. The SNP showed the strongest effect on expressed transcripts for *AL359058* (p = 5.1×10^−10^, explained variance = 4.2%) and for *LRRC61* (p = 7.8×10^−9^, explained variance = 3.7%). The transcripts were not correlated (r^2^ = 0.004, p = 0.54) (Table 3). Both transcripts map within the chromosomal region harbouring variants with the lowest p-values in the genome wide meta-analysis (about 26 kB from the start site of AL359058 and about 22 kB from the start site of *LRRC61*) suggesting *cis* regulation. *RARRES2* transcripts were not expressed in PBMCs in our study.

**Table 3:** eQTL analysis for rs7806429 in PBMCs of the Sorbs cohort.

		Association of rs7806429 with transcript			Association of transcript with circulating chemerin levels			Test for causality
Transcript ID Illumina	Gene Symbol	*β* (SE)	p-value	Explained variance	*β* (SE)	p-value	Explained variance	p-value
ILMN_1867457	AL359058	−0.039	5.11×10^−10^	4.2%	0.166	0.1	0.35%	0.9
		(0.00625)			(0.101)			
ILMN_1669722	LRRC61	−0.038	7.78×10^−9^	3.7%	−0.016	0.86	0.00%	0.89
		(0.00660)			(0.0943)			

Although we observed the above mentioned strong *cis*-effects of our lead SNP, none of the transcripts was associated with chemerin levels. Consequently, no evidence for a chain of causation of the form rs7806429→gene-expression→serum chemerin was found (Table 3 and S2 Figure).

Moreover, we extensively searched throughout available online resources of known eQTLs. Results are summarized in S5 Table. It revealed that rs7806429 is in linkage disequilibrium (r^2^>0.5) with several cis-eQTLs in various tissues including RARRES2 in brain [16] and lymphoblastoid cell lines [17].

#### Adipose tissue

We analysed the association of rs7806429 (SNP with the strongest evidence for association with chemerin) with *RARRES2* transcript levels in visceral and subcutaneous adipose tissue. The SNP rs7806429 was moderately associated with mRNA expression of *RARRES2* in visceral adipose tissue (Table 4). The association has been driven by SNP effects in women, whereas no association was found in men (Table 4). Sex-interaction was not significant. Of note, consistent with correlation analyses with circulating chemerin, *RARRES2* mRNA levels correlated with anthropometric traits (% body fat), as well as parameters of glucose homeostasis (fasting plasma glucose, glucose infusion rate) and lipid metabolism (LDL-cholesterol) (all p<0.05 after adjusting for age, sex and BMI) (S3 Table).

**Table 4:** Association of rs7806429 with *RARRES2* expression in subcutaneous and visceral adipose tissue.

Chemerin/18S rRNA	Female			Male			Total cohort			
	N	p-value	*β*	N	p-value	*β*	N	p-value	*β*	p-value (sex-interaction)
**SC adipose tissue**	114	0.927	−0.005	51	0.574	0.082	165	0.932	0.004	0.624
**VIS adipose tissue**	114	**0.010**	−0.139	49	0.652	0.067	163	0.052	−0.095	0.085
**Ratio VIS/SC adipose tissue**	112	**0.006**	−0.142	49	0.957	−0.008	161	**0.015**	−0.106	0.162

Linear regression analyses adjusted for age and BMI (and sex for the total cohort). Subjects with T2D were not included in the analyses.

SC: subcutaneous; VIS: visceral; β: beta.

## Discussion

Chemerin is an adipokine proposed to link obesity and chronic inflammation of adipose tissue. Consistent with previous reports [1], [2], serum chemerin concentrations were positively correlated with BMI and % body fat in our study. Furthermore, chemerin levels correlated with elevated fasting insulin, HOMA-IR and AUC_glucose_. Although serum chemerin levels are heritable, with estimates of 0.25 in Mexican-Americans [8], studies aimed at identification of genetic factors explaining the variability in circulating chemerin levels are lacking. Here, we conducted a meta-analysis of GWAS for serum chemerin in three independent cohorts from Europe (Germany and Sweden/Finland). The heritability of chemerin in the Sorbs cohort was re-estimated to be 16.2% which is close to the above mentioned estimate. We found 30 SNPs within the *RARRES2-LRRC61* locus on chromosome 7 which reached genome-wide significance levels in the meta-analysis. The strongest evidence for association was observed at rs7806429, which represents a cluster of SNPs in pairwise LD.

The results of the subgroup analyses in males and females were consistent with findings in the total cohort. No significant SNP-sex interaction was observed. Our lead SNP rs7806429 is in LD with rs10278590 (r^2^ = 0.64 in the Sorbs cohort) reported to be associated with visceral fat mass in non-obese subjects [9] but not with rs17173608 (r^2^ = 0.049 in the Sorbs cohort) reported to be associated with the incidence of metabolic syndrome [10]. Mendelian randomization analysis suggests a causal relationship between chemerin and adiponectin in the Sorbs cohort, which may appear plausible when considering the potential role of chemerin in regulation of adipogenesis [5]. Yet, we see these data with caution since only nominal significance was achieved for the causality test, i.e. results are not robust against multiple testing correction.

Noteworthy, despite missing genome-wide significance levels for association with chemerin, the present GWAS revealed additional variants on chrs. 3 (lead SNP: rs2594989 in *ATG7*) and 15 (lead SNP: rs8027521) definitely deserving further consideration. The first locus showed consistent effects across all studies. It includes the *ATG7* which seems to be an appealing candidate for metabolic studies when considering the emerging evidence for the implication of autophagy in the regulation of adipose tissue and beta cell functions [18]. Although the precise mechanism by which autophagy regulates adipogenesis is unknown, it has been shown that Atg7−/− animals have smaller white adipose tissue depots and adipocyte-specific knockdown of Atg7 lead to the development of brown like adipose tissue [19], [20]. One might therefore hypothesise that any developmental disturbance in autophagy may affect adipose tissue mass and homeostasis. Thus, alteration in autophagy caused by genetic variants in autophagy genes (e.g. *ATG7*) might result in adverse adipogenesis which consequently might influence expression of adipokines such as chemerin.

In addition, we tested the association of SNPs reported by Bozaoglu et al. [8] with chemerin levels in our meta-analysis (S4 Table). Five out of the seven reported SNPs were included in our meta-analysis. For the other two SNPs we analysed adequate proxies, namely rs12534101 for rs11971186 (r^2^ = 0.99) and rs6701545 for rs4446959 (r^2^ = 1.0). We observed only nominal significance of rs1405069 in the Sorbs cohort and of rs347344 in the PPP cohort, the latter SNP having discordant direction of effect between cohorts. In the meta-analysis, we observed only nominal significance for rs1405069 with concordant direction of effects between cohorts. Hence, we could not convincingly replicate the hits reported by Bozaoglu et al. These inconsistencies between the studies could possibly be attributed to different ethnicities of the studied cohorts (Mexican-Americans vs. Europeans) as well as limited sample size of the previous GWAS by Bozaoglu et al which only included 523 individuals [8].

To functionally support the present GWAS findings, we sought to elucidate possible molecular mechanisms underlying the SNP associations with serum chemerin. An eQTL analysis in PBMCs revealed a strong association between rs7806429 and mRNA levels of *AL359058* and *LRRC61* (also known as *FLJ31392, HSPC295, MGC3036*). Both transcripts map within the chromosomal region harbouring variants with lowest p-values in the chemerin GWAS, thus suggesting *cis*-regulation of the expression of corresponding genes. Nevertheless, none of the transcripts correlated with chemerin levels, i.e. the association between rs7806429 and serum chemerin does not seem to be mediated by the mRNA expression of *AL359058* or *LRRC61* in PBMCs. Unfortunately, we could not analyse possible SNP associations with *RARRES2* transcripts since these were not expressed in PBMCs. However, our top-SNP is in linkage disequilibrium with variants showing eQTL effects of *RARRES2* in other tissues such as brain and lymphoblastoid cell lines [16], [17]. In this line, rs7806429 was associated with mRNA expression of *RARRES2* in visceral adipose tissue of female participants from the Leipzig cohort. We only detected significant associations in women. Hence, this result may at least in part suggest that the observed SNP-chemerin association could be mediated by the role of the variant in transcription control. It has to be acknowledged though, that serum chemerin was not available in the Leipzig cohort, i.e. no direct evidence for correlation between adipose mRNA expression and serum chemerin could be tested. Nevertheless, consistent correlations of metabolic parameters with both, *RARRES2* mRNA expression in the Leipzig cohort as well as serum chemerin in the Sorbs cohort suggests a relationship of *RARRES2* expression in fat tissue and circulating chemerin levels which could explain the observed SNP-chemerin associations. Further support comes from Min et al. who employed a whole-genome expression and genotype profiling on abdominal adipose tissue and found that rs10282458, an eSNP affecting expression of *RARRES2*, was associated with BMI [11]. It is noteworthy that rs7806429, the lead SNP from our study, is in high LD (r^2^>0.7) with rs10282458, which however, was not included in our meta-analysis due to quality issues. On the other side, we further checked for potential associations in publically available GWAS datasets from GIANT consortium (http://www.broadinstitute.org/collaboration/giant/index.php/GIANT_consortium) and found rs7806429 being nominally associated with BMI (p = 0.01; N = 123,835). In contrast, no associations were observed for glucose and insulin related traits in the GWAS dataset from MAGIC consortium (http://www.magicinvestigators.org).

Moreover, functional consequences of the associated SNPs were predicted using the RegulomeDB [21]. Including all 26 SNPs in strong LD with rs7806429 (based on r2>0.8 in HapMap CEU population), we identified 17 variants reaching a score of 1, suggesting that these polymorphisms might explain an eQTL or affect corresponding transcription factor binding sites. For example, as shown for rs17173617, the reported eQTL associates with the expression of *LRRC61*, *RARRES2* and *C7orf29* in lymphoblastoid cell types [17], [22]. Regarding to transcription factor binding sites, it is noteworthy that rs2108854 maps within the FOXP1 [23], rs3735171 within the AP-4 [23], [24] or Brn-2 and rs11769348 within the NFE2L2 binding motif [24]. These data strongly suggest causative variants within the chemerin-associated locus and warrant further in-depth functional analyses to elucidate molecular mechanisms underlying the observed associations.

Finally, we would like to note that it may not appear surprising that variants associated with circulating chemerin map within a locus including the *RARRES2* which itself encodes chemerin. However, history of GWAS showed that biologically plausible genes do not necessarily point towards easily identifiable risk variants. Indeed, our locus was not discovered by the previous GWAS for chemerin [8].

In conclusion, the present meta-analysis of GWAS for serum chemerin levels is the first study to demonstrate robust SNP-associations reaching genome-wide significance and highlights the role of genetic variation in the *RARRES2* in the regulation of circulating chemerin concentrations possibly caused by altered gene expression in fat tissue.

## Materials and Methods

### Subjects

#### Sorbs cohort

The cohort was recruited from the self-contained Sorbs population in Germany [25]–[28]. Extensive phenotyping included standardized questionnaires to assess past medical history and family history, collection of anthropometric data (weight, height, waist-to-hip ratio (WHR)), and an oral glucose tolerance test (OGTT). Glucose was assessed by the Hexokinase method (Automated Analyser Modular, Roche Diagnostics, Mannheim, Germany) and serum insulin was measured using the AutoDELFIA Insulin assay (PerkinElmer Life and Analytical Sciences, Turku, Finland). Chemerin serum concentrations in the three cohorts were determined using a commercially available ELISA kit (Human Chemerin ELISA, Biovendor, Heidelberg, Germany) according to the manufacturer's instructions. Blood samples were taken in the morning after an overnight fast and stored at −80°C until analyses. The sensitivity of the chemerin ELISA assay was 0.1 ng/ml. The degree of precision of the ELISA system in terms of coefficient of variance (%) of intra-assay was 6% (within-run, n = 8), that of inter-assays was 7.6% (run-to-run, n = 6).

In the current study, the cohort included 824 Sorbs without type 2 diabetes, with complete genotype, covariate and phenotype information (S1 Table). The study was approved by the ethics committee of the University of Leipzig and all subjects gave written informed consent before taking part in the study.

#### KORA

The Cooperative Health Research in the Region of Augsburg (KORA) study is a series of independent population-based epidemiological surveys and follow-up studies of participants living in the region of Augsburg, Southern Germany. All participants are residents of German nationality identified through registration. Informed consent has been given by all participants [29] and the study has been approved by the local ethics committee. The present study includes data of the KORA follow-up study F4 (2006–2008). For the genome-wide association study, we genotyped 1,814 randomly selected participants of KORA F4. 1630 subjects without type 2 diabetes were included in the present study. Chemerin serum concentrations were determined using a commercially available ELISA (Human Chemerin ELISA, Biovendor, Heidelberg, Germany) as described above. Detailed phenotypic information is provided in S1 Table.

#### Prevalence, Prediction and Prevention of diabetes (PPP)-Botnia Study

The Prevalence, Prediction and Prevention of diabetes (PPP)–Botnia Study is a population-based study from the Botnia region of western Finland [30]. In the present study, 337 subjects with normal glucose tolerance from PPP that also participated in the Diabetes Genetics Initiative (DGI) were included [31]. Chemerin serum concentrations were determined using a commercially available ELISA kit (Human Chemerin ELISA, Biovendor, Heidelberg, Germany) as described above.

Participants gave their written informed consent and the study protocol was approved by the Ethics Committee of Helsinki University Hospital, Finland.

#### LIFE Leipzig Heart Study

This is an observational study recruiting individuals with suspected coronary artery disease due to clinical symptoms (non-invasive testing), with stable left main coronary artery disease or myocardial infarction. Details of the study can be found elsewhere [15]. The study meets the ethical standards of the Declaration of Helsinki. It has been approved by the Ethics Committee of the Medical Faculty of the University Leipzig, Germany (Reg. No 276-2005) and is registered at ClinicalTrials.gov (NCT00497887).

In the present study, 967 nondiabetic subjects were included. Chemerin was measured by a commercially available ELISA (Mediagnost, Reutlingen,Germany) adapted on the DSX automatic ELISA test system. The analytical sensitivity was 0.005 ng/mL. Intra and inter assay coefficient of variation were found to be 4.9% at a concentration of 61.8 ng/mL and 6.3% at a concentration of 144 ng/mL (n = 15) chemerin.

Written informed consent including agreement with genetic analyses was obtained from all participants enrolled in the study.

### Leipzig adipose tissue samples

Paired samples of visceral (vis) and subcutaneous (sc) adipose tissue were obtained from 636 Caucasian men (N = 203) and women (N = 433), undergoing open abdominal surgery. The subjects had a mean age of 50±14 years and a mean BMI of 43.9±12.9 kg/m^2^ (women: 49±13 years and 44.5±12.3 kg/m^2^; men: 53±15 years and 42.8±14.2 kg/m^2^). All subjects maintained a stable weight (within 2% of the body weight) for at least 3 months before surgery. Patients with severe conditions including generalized inflammation or end-stage malignant diseases were excluded from the study. Samples of vis and sc adipose tissue were immediately frozen in liquid nitrogen after explantation. An OGTT was performed after an overnight fast with a 75 g standardized glucose solution (Glucodex Solution 75 g; Merieux, Montreal, Canada). In addition to the above mentioned clinical parameters, abdominal vis and sc fat area were calculated using computed tomography scans at the level of L4-L5, and percentage body fat was measured by dual-energy X-ray absorptiometry (DEXA).

The ethics committee at the Medical Faculty of the University of Leipzig specifically approved this study, and all subjects gave written informed consent before taking part in the study.

### Genotyping, quality control and genotype imputations

#### Sorbs cohort

Subjects were genotyped either by 500 K Affymetrix GeneChip or by Affymetrix Genome-Wide Human SNP Array 6.0. The BRLMM algorithm (Affymetrix, Inc) was applied for the 500 K array and the Birdseed algorithm was applied for the Genome-Wide Human SNP Array 6.0. Quality control of samples was performed as described in Gross et al. [26], resulting in N = 977 individuals with genotypes of good quality (N = 483 genotyped with the 500 K assay, N = 494 genotyped with the 6.0 assay). Genotype imputation was performed separately for individuals genotyped with the two different assays. No prior SNP filtering was performed. Imputation was performed with IMPUTE v2.1.2 (http://mathgen.stats.ox.ac.uk/impute/impute_v2.html) using HapMap2 CEU, Release 24, dbSNP-build 126, NCBI 36 as the reference panel. To detect ethnical outliers, we applied a ‘drop one in’ procedure to avoid bias by the relatedness structure within the Sorbs cohort [28]. We performed one PCA for each Sorbian individual together with 50 most unrelated HapMap CEU individuals based on genotype data as explained in Gross et al [26]. Resulting eigenvectors of CEU individuals were averaged over all iterations. Individuals were considered as ethnical outliers if the distance from the mean of the respective eigenvector of at least one of the first 10 eigenvectors exceeds 6 sd. After application of filtering, three individuals were discarded from association analysis (final N = 824). Details are provided in S1 Table.

#### KORA

Subjects were genotyped at the Helmholtz Genome Analysis Center using Affymetrix 6.0 arrays. The Birdseed 2 calling algorithm was applied. Imputations were performed with IMPUTE v0.4.2 on HapMap Phase 2 CEU as a reference panel (details see S1 Table).

#### PPP

Subjects were genotyped at the Broad Institute using the Affymetrix GeneChip Human Mapping 500 K Array Set. The BRLMM algorithm was applied. Imputations were performed with MACH on HapMap Phase 2 CEU as the reference panel (details see S1 Table).

#### LIFE Leipzig Heart Study

Genotyping was performed using the Affymetrix Axiom Technology with custom option (Axiom-CADLIFE). Genotype calling was performed with Affymetrix Power Tools version 1.12. Sample quality control comprised call rate (>97%), hetero- or homozygosity excess (outliers of mean squared differences of observed and expected genotypes), sex-mismatch, cryptic relatedness, and outliers of principal components analysis (6SD criterion of Eigenstrat software) [32]. Prior to imputation, low quality SNPs defined by low call-rate (<90% plate-wise call rate corresponding to <94.2% overall call-rate), deviation from Hardy-Weinberg equilibrium (p<10^−6^) or plate-association (p<10^−7^) were filtered. Individuals were imputed at the 1000Genomes reference phase 1, release 3 (http://mathgen.stats.ox.ac.uk/impute/data_download_1000G_phase1_integrated.html) using SHAPEIT v2 and IMPUTE 2.3.0. For post-imputation quality control we filtered SNP with minor allele frequency<1% or info-score<0.3.

### Genotyping of rs7806429 in the Leipzig cohort

*De novo* genotyping of the SNP rs7806429 was done using the TaqMan SNP Genotyping assays according to the manufacturer's protocol (Applied Biosystems, Inc., Foster City, CA). To validate the reproducibility of genotyping, a random subset (about 5%) of the sample was re-genotyped; all genotypes matched initial designated genotypes. No deviations from HWE were observed (p>0.1).

### Association studies and meta-analysis

#### Association between circulating chemerin levels and anthropometric traits

Before analyses, all non-normally distributed parameters were logarithmically transformed (% body fat, BMI, WHR, fasting insulin, HOMA-IR, Stumvoll index, AUC glucose, AFABP, progranulin, vaspin, adiponectin, chemerin, LDL cholesterol, glomerular filtration rate). Linear regression models adjusted for age, sex, and BMI (only age and sex for BMI analysis) were calculated using SPSS software version 20.0 (SPSS, Chicago, IL).

#### Genome wide association analysis

Chemerin serum levels were log-transformed prior to analysis. Genetic associations were assessed using linear regression analyses of allele doses adjusting for age, sex and BMI. In the Sorbs cohort, we additionally adjusted for relatedness using mixed effect models (function “polygenic” of the “GenABEL” package of R) [12], [13]. We also performed sub-group analyses in males and females. Meta-analysis was performed for SNPs fulfilling the quality control criteria in all three cohorts (see section “Genotyping, quality control and genotype imputations”). SNPs with allele frequencies differing by more than 20% between cohorts were discarded. According to this criterion, a total of 2,023,499 SNPs were analysed. Prior to meta-analysis, effect sizes of single studies were corrected for inflation (Sorbs cohort: λ = 1.014, KORA: λ = 0.99, PPP: λ = 0.99). Standardized effects of single studies were combined using fixed and random effects models (function “metagen” of the “meta” package of R). Heterogeneity between studies was assessed by Q-statistics.

### Functional consequences of the associated SNPs using RegulomeDB

Potential functional consequences of the associated SNPs were analysed using the RegulomeDB [21]. Briefly, RegulomeDB is a database that annotates SNPs with known and predicted regulatory elements in the human genome, such as DNAase hypersensitivity, binding sites of transcription factors, and promoter regions.

### Mendelian randomisation

Finally, a Mendelian randomization analysis was performed using our top-associated SNP as an instrumental variable [33]. This analysis aimed at establishing causal links between chemerin and other parameters of obesity and the metabolic syndrome as presented in Table 1.

### Replication analysis

Replication of top-SNPs was performed in samples of the LIFE Leipzig Heart Study. Top-SNPs to be replicated were retrieved from these data by searching for well-imputed proxies using SNAP (https://www.broadinstitute.org/mpg/snap/ldsearch.php).

For a total of 967 non-diabetic individuals, both, chemerin measurements with adequate covariate information and SNP data were available. For replication analysis, we analysed additive models of chemerin-SNP associations adjusting for age, sex and BMI as in the cohorts of the meta-analysis. Taking into account the characteristics of the LIFE LeipzigHeart study population, we also adjusted for fasting status and disease status (coronary artery disease case/control status).

### mRNA expression studies

#### RNA expression profiling in PBMCs in the Sorbs cohort

PBMCs were extracted from blood samples collected in VACUTAINER CPT (Cell Preparation Tubes) containing sodium heparin as the anti-coagulant according to the manufacturer's protocol (BD, Franklin Lakes, NJ). RNA from PBMCs was extracted using the TRIzol protocol (Thermo Fisher Scientific). DNase I digestion of the RNA samples was performed with subsequent RNA clean-up using the RNeasy MinElute Cleanup Kit (Qiagen, Hilden, Germany). RNA integrity and concentration were examined using an Agilent 2100 Bioanalyzer (Agilent Technologies, Palo Alto, CA, USA) using the RNA 6.000 LabChip Kit (Agilent Technologies) according to the manufacturers' instructions. Illumina GeneChip analyses were conducted at the microarray core facility of the Interdisciplinary Centre for Clinical Research (ICCR) in Leipzig (Dr. Krohn, Faculty of Medicine, University of Leipzig). 250 ng of total RNA was reverse transcribed cDNA (Target Amp labelling kit (Illumina, SanDiego, CA, USA and Superscript III, Life Technologies, Gaithersburg, MD, USA), which was further synthesized to cRNA by *in vitro* transcription (Superscript III, Life Technologies, Gaithersburg, MD, USA and Target Amp labelling kit, Illumina, SanDiego, CA, USA). Subsequently, unincorporated nucleotides were removed using the RNeasy kit (QIAGEN, Hilden, Germany) and the cRNA was hybridized to the Illumina Human HT-12 v4 Bead Chip. Washing and staining of the probe array were performed according to the manufacturers' instructions. The arrays were scanned using an Illumina High Scan SQ.

For data analysis, probe set fluorescence intensities were extracted for available transcripts and were scaled to normalize data for inter-array comparison using High Control software (Illumina, San Diego, CA, USA).

### Pre-processing of RNA microarray data

Pre-processing of RNA microarray data relied on the intensities of 47,323 transcripts derived from Illumina BeadStudio without background correction and normalization measured in 1,029 individuals of the Sorbs cohort. Steps for pre-processing comprised 1. filtering of individuals with atypical low number of expressed genes (median - 3 interquartile ranges (IQR) of the cohort's values), 2. quantile normalization and log2- transformation, 3. filtering individuals with atypical gene-expression profiles (Euclidian distance to average expression larger than median +3 IQR), 4. filtering individuals with atypical values of internal quality parameters (quantified as Mahalanobis distance of quality control probes included on the HT-12 v4 chip by Illumina, individuals having a larger value than median +3 IQR of this measure were excluded), 5. correcting for batch effects on the basis of hybridisation chip numbers using Empirical Bayes estimates [34], 6. linear adjustment for age, sex, and lymphocyte and monocyte cell counts. A total of 924 individuals fulfilled all quality criteria. For 898 of these, SNP array data were also available for eQTL analyses.

### Gene-expression analyses of PBMCs in the Sorbs cohort

To provide functional evidence for the SNPs with the strongest evidence for association in the meta-analysis, we performed eQTL analyses in PBMCs including all SNPs reaching genome-wide significant association with chemerin serum levels. Moreover, we examined potential correlations between the corresponding regulated transcripts and chemerin levels. Finally, we tested for causal relationships between top-SNPs, gene-expression and chemerin levels by comparing effect sizes (beta-coefficients) of SNPs between the two linear models: “chemerin depending on SNP” and “chemerin depending on regulated gene-expression and SNP”.

### Human adipose tissue expression of RARRES2 in the Leipzig cohort

To measure human adipose tissue expression of *RARRES2* in the Leipzig cohort, total RNA was isolated from paired subcutaneous and visceral adipose tissue samples using TRIzol (Life Technologies, Grand Island, NY), and 1 µg RNA was reversely transcribed using standard reagents (Life Technologies). *RARRES2* mRNA expression was measured by quantitative real-time RT-PCR using TaqMan methodology, and fluorescence was detected on an ABI PRISM 7500 sequence detector according to the manufacturer's instructions (Applied Biosystems, Darmstadt, Germany). Human *RARRES2* mRNA expression was calculated relative to the mRNA expression of *HPRT* and *18S rRNA*, determined by a premixed assay on demand (PE Biosystems, Darmstadt, Germany). The specificity of the PCR was further verified by agarose gel electrophoresis.

## Supporting Information

S1 Figure Forest plot for associations with s7806429.
(PPTX)

S2 Figure Gene-expression and rs7806429.
(PPTX)

S1 Table Cohort description and methods.
(XLS)

S2 Table Association of SNPs with chemerin.
(XLSX)

S3 Table Chemerin mRNA expression and metabolic traits.
(XLS)

S4 Table Association of SNPs proposed by Bozaoglu et al.
(XLS)

S5 Table Annotated eQTLs (GTEx).
(XLSX)

S6 Table Mendelian randomization.
(XLSX)

S7 Table Replication of association signals in the LIFE Leipzig Heart Study.
(XLSX)
