# Supplementary figures and images for: Genome Wide Meta-analysis Highlights the Role of Genetic Variation in RARRES2 in the Regulation of Circulating Serum Chemerin
Source: PLoS Genet. 2014 Dec 18;10(12):e1004854. doi: 10.1371/journal.pgen.1004854 (PMC4270463; doi:10.1371/journal.pgen.1004854)

## Slide 1
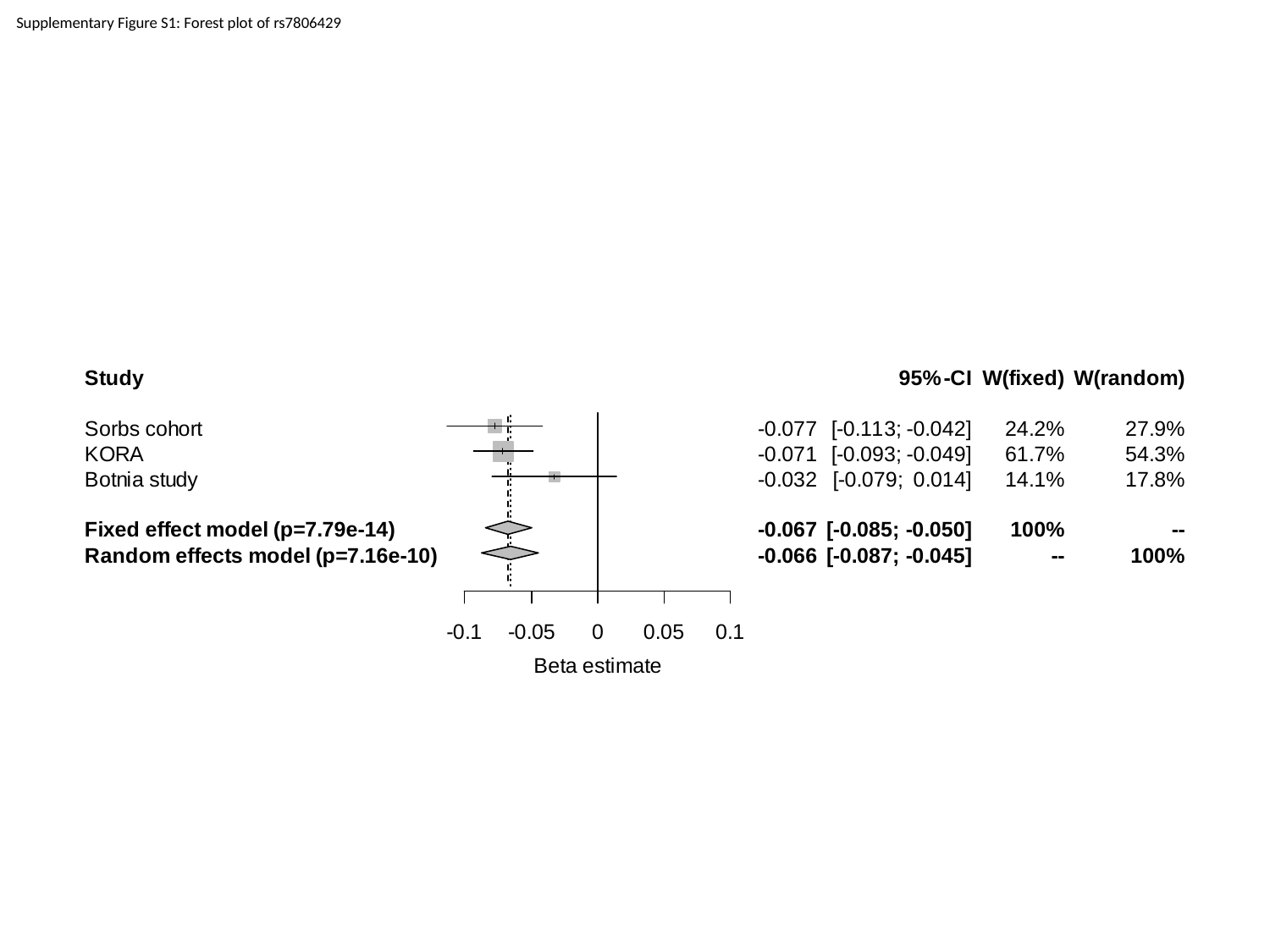

Supplementary Figure S1: Forest plot of rs7806429

Supplement: S1 Figure — Forest plot for associations with s7806429. (PPTX) [file pgen.1004854.s001.pptx]
