# Supplementary material for: Genome Wide Meta-analysis Highlights the Role of Genetic Variation in RARRES2 in the Regulation of Circulating Serum Chemerin
Source: PLoS Genet. 2014 Dec 18;10(12):e1004854. doi: 10.1371/journal.pgen.1004854 (PMC4270463; doi:10.1371/journal.pgen.1004854)
Supplement: S2 Figure — Gene-expression and rs7806429. (PPTX) [file pgen.1004854.s002.pptx]

## Slide 1
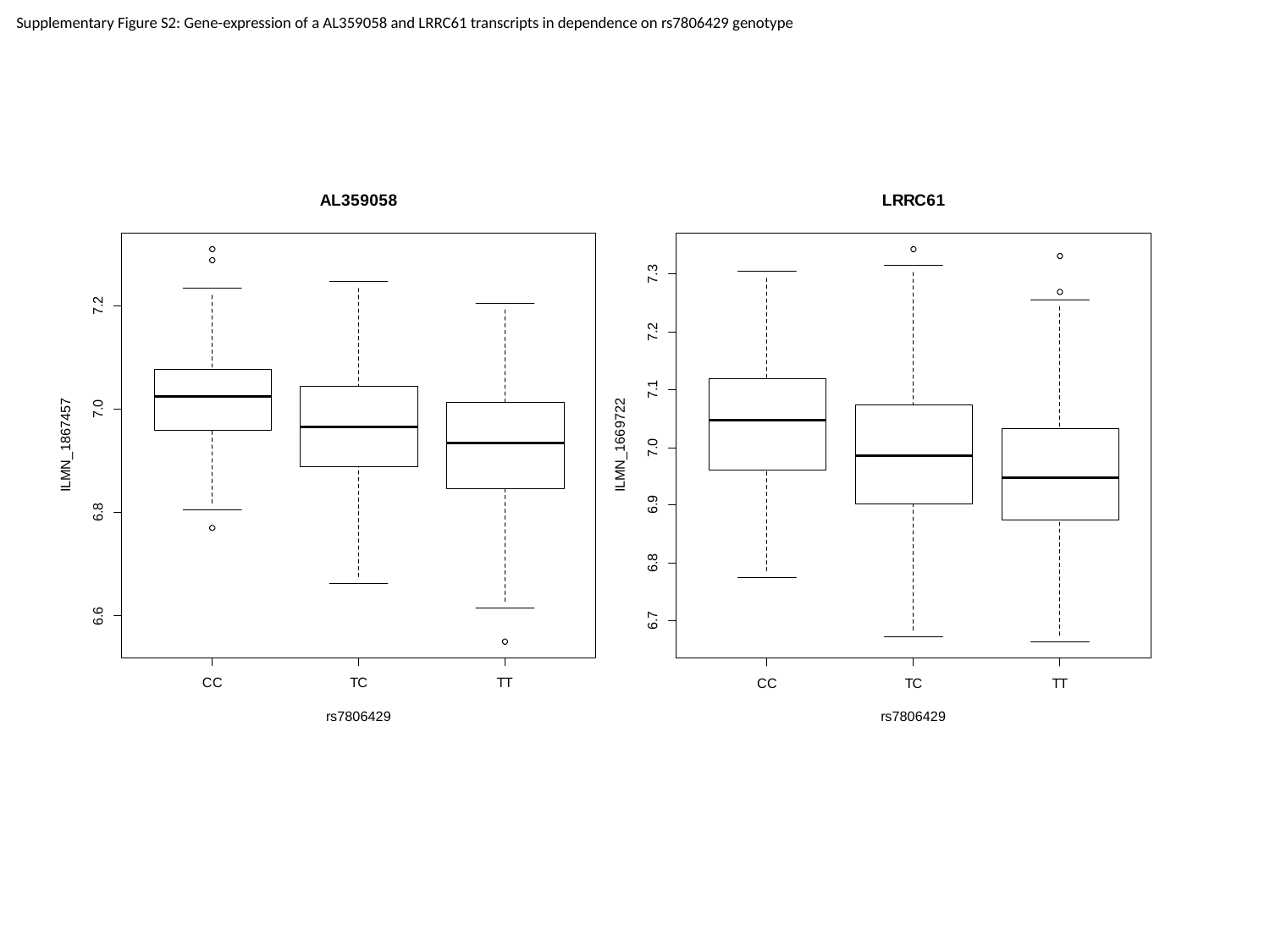

Supplementary Figure S2: Gene-expression of a AL359058 and LRRC61 transcripts in dependence on rs7806429 genotype
